# Folic Acid Attenuates High-Fat Diet-Induced Osteoporosis Through the AMPK Signaling Pathway

**DOI:** 10.3389/fcell.2021.791880

**Published:** 2022-01-03

**Authors:** Haiting He, Yaxi Zhang, Yue Sun, Yanwei Zhang, Jingjing Xu, Yuzhen Yang, Jihua Chen

**Affiliations:** Department of Nutrition Science and Food Hygiene, Xiangya School of Public Health, Central South University, Changsha, China

**Keywords:** folic acid, osteoporosis, high-fat diet, lipid metabolism, AMPK

## Abstract

**Objective:** Obesity caused by a high-fat diet (HFD) will expand adipose tissue and cause chronic low-grade systemic inflammation, leading to osteoporosis. Folic acid (FA) is a water-soluble vitamin that plays an essential role in regulating blood lipids and antioxidants. However, the effects and underlying mechanisms of FA in osteoporosis induced by an HFD remain poorly understood. This study aimed to investigate the effect of FA on bone health by using HFD-induced osteoporosis mice.

**Materials and Methods:** Mice were fed a normal diet, HFD or an HFD supplemented with FA (20 μg/ml in drinking water) for 16 weeks. Throughout the 16 weeks study period, the rats were weighed once every week. GTT, ITT and lipid indexes were detected to evaluate the effects of FA on lipid metabolism in the HFD-fed mice. Morphological and structural changes of the femur and tibial bone were observed using micro-CT, HE staining and bone conversion parameters. The expression of MDA, SOD and inflammatory factors were detected to evaluate the effects of FA on oxidative stress and inflammatory response in the HFD-fed mice. Quantitative real-time PCR and Western blot (WB) were used to investigate the AMPK signaling pathway.

**Results:** After the intervention of FA, the body fat rate of obese mice was reduced, and related metabolic disorders such as insulin resistance, hyperlipidemia, and systemic inflammation were alleviated. In correlation with those modifications, FA attenuated bone loss and improved bone microarchitecture, accompanied the number of osteoclasts and adipocytes decreased. Furthermore, FA promoted the phosphorylation of AMPK, thereby promoting the expression of Carnitine palmitoyltransferase 1 (CPT1), nuclear factor erythroid-2 related factor 2 (Nrf2) and antioxidant enzymes.

**Conclusion:** These findings suggest that FA may modulate lipid metabolism and oxidative stress responses activating the AMPK signaling pathway, thereby alleviating HFD-induced osteoporosis. The results from our study provide experimental evidence to prevent HFD-induced osteoporosis.

## Introduction

Obesity caused by an HFD will dilate adipose tissue and lead to chronic low-grade systemic inflammation. Ectopic adipose cells accumulate in the bone marrow cavity, impair bone regeneration and lead to osteoporosis (Halade et al., 2010). Bone marrow is the only tissue where adipocytes and bone cells directly interact (Horowitz and Tommasini, 2018). Studies have found that osteoporosis or bone mass loss often coexists with lipid metabolism disorders (Tian and Yu, 2015). When the number and volume of adipocytes in the bone marrow continue to increase, the microenvironment in the bone marrow cavity changes, causing the bone marrow lipid metabolism disorder, the differentiation of bone marrow mesenchymal stem cells (BMSCs) into osteoblasts decreases, the formation and function activation of osteoclasts increase, and the total amount of excessive bone loss, eventually leading to osteoporosis (Hu et al., 2018). Studies have shown that obesity is often accompanied by abnormal secretion of adipokines (Fasshauer and Blüher, 2015). Abnormal production of adipokines and activation of some pro-inflammatory signaling pathways in obese patients can induce accelerated inflammation. Inflammation activates various immune cells to produce many free radicals, aggravating oxidative stress, and oxidative stress (OS) also plays a crucial role in the pathogenesis of osteoporosis (Saltiel and Olefsky, 2017; Geng et al., 2019).

AMP-activated protein kinase (AMPK) is the regulatory switch of energy metabolism in organisms, and it plays a crucial role in lipid metabolism regulation (Hardie, 2015). Several studies have shown that it promotes the oxidation and decomposition of fatty acids, inhibiting lipid synthesis and other processes from inhibiting lipid accumulation (Kim et al., 2016; Garcia et al., 2019). In addition, AMPK can be activated under oxidative stress. The activated AMPK can produce ATP by promoting insulin sensitivity, fatty acid oxidation, and mitochondrial biosynthesis, thus reducing oxidative stress damage (Herzig and Shaw, 2018). Previous studies have reported that activating the AMPK signaling pathway can induce mitochondrial autophagy of bone marrow mesenchymal stem cells to inhibit intracellular oxidative stress and improve osteoporosis (Chen et al., 2021).

FA is a water-soluble vitamin widely found in fresh fruits, vegetables, and meat. FA is highly safe, preventing DNA damage, reducing oxidative stress and cell apoptosis. It has no significant side effects in the long-term use of small doses (Field and Stover, 2018). Studies have shown that FA is an effective free radical scavenger, which can inhibit lipid peroxidation and reduce the production of malonic acid (MDA) (Li et al., 2021). FA deficiency can cause an increase in homocysteine and a decrease in methionine, which further increases liver lipid synthesis and induces lipid accumulation in liver cells (Rizki et al., 2006). In addition, studies have shown that low FA levels can significantly reduce trabecular bone thickness and trabecular bone number, and there may be a direct relationship between FA and bone mineral density (Holstein et al., 2009). These results suggest that FA may prevent bone loss, but FA’s exact effect and mechanism on bone metabolism remain unclear.

Therefore, our current study aims to explore whether FA could affect bone health and related mechanisms of HFD-fed mice by regulating lipid metabolism and oxidative stress by using the AMPK signaling pathway as the entry point to provide valuable information-theoretical evidence for nutritional intervention strategies of HFD-induced osteoporosis.

## Materials and Methods

### Chemicals and Reagents

FA was purchased from Sigma-Aldrich (St. Louis, MO, United States). Alanine aminotransferase (ALT, C009-2-1), aspartate aminotransferase (AST, C010-2-1), triacylglycerol (TG, A110-1-1), total cholesterol (TC, A111-1-1), high-density lipoprotein cholesterol (HDL-C, A112-1-1), low-density lipoprotein cholesterol (LDL-C, A113-1-1), superoxide dismutase (SOD, A001-3-2) and malondialdehyde (MDA, A003-1-2) assay kits were obtained from Jiancheng Bioengineering Institute (Nanjing, China). The ELISA kits for detecting tumor necrosis factor-α (TNF-α, RK00027), interleukin-6 (IL-6, RK00008), *β*-actin (AC026), acetyl-CoA carboxylase (ACC, A15606) and carnitine palmitoyltransferase 1 (CPT1, A5307) were purchased from Abclonal (Boston, United States). EDTA decalcified fluid (AR1071) was purchased from Boster (California, United States). Hematoxylin-eosin (G1005) was purchased from Servicebio Biotechnology Co., Ltd. (Wuhan, China), phenylmethyl sulfonyl fluoride (PMSF, ST506) was purchased from Beyotime (Shanghai, China). Minute™ Bone Tissue Total Protein Extraction Kit was purchased from Invent Biotechnologies, Inc.(Eden Prairie, United States). FastPure^®^ Cell/Tissue Total RNA Isolation Kit and ChamQ universal SYBR qPCR Master Mix(Q711-02) were purchased from Vazyme Biotech Co.,Ltd. (Nanjing, China). RevertAid First Strand cDNA Synthesis Kit was purchased from Thermo Scientific (Waltham Mass, United States).The primary antibodies against heme oxygenase-1 (HO-1, SC-390991) and glutamate-cysteine ligase modifier subunit (GCLM, SC-55586) were purchased from Santa Cruz Biotechnology (California, United States). The primary antibodies against nuclear factor-E2-related factor 2 (Nrf2, 12721S) was purchased from Cell Signaling Technology (MA, United States). The primary antibodies against NAD(P)H quinone dehydrogenase 1(NQO1, ab28947) was purchased from Abcam (Cambridge, United Kingdom). The secondary antibody horseradish peroxidase (HRP) Goat Anti-Rabbit IgG (H + L) (IH-0011) and horseradish peroxidase (HRP) Goat Anti-Mouse IgG (IH-0032) were purchased from Ding Guo Changsheng Biotechnology Co. Ltd. (Beijing, China).

### Animals and Diet

Thirty male C57/BL6J mice aged 6–8 weeks were purchased from Beijing Weitong Lihua Laboratory Animal Technology Co., Ltd. [animal license number SCXK (Beijing, China) 2016-0006]. They were kept under laboratory conditions at a room temperature of 22 ± 2°C, 50%–60% relative humidity, and 12 h light-dark cycle. After all the mice were fed adaptively for 2 weeks, the control group (*n* = 10) were fed a regular chow diet (10% kcal of fat, 70% kcal of carbohydrate, 20% kcal protein, D12450J, Research Diets)), the others were fed HFD (60% kcal of fat, 20% kcal of carbohydrate, 20% kcal of protein, D12492, Research Diets), and the HFD + FA group were given FA supplementation at a concentration of 20 μg/ml. All mice were fed for 16 weeks. The way mice take FA is to drink water with FA (Li et al., 2018). The mice in each group ate and drank freely, the researchers recorded their food and water intake every day and calculated the daily folate intake of the mice, and weighed them once a week. All animal experiments were approved by the Animal Ethics Committee of Central South University, and the submission number is XYGW-2020-28.

### Glucose Tolerance Test and Insulin Tolerance Test

On the 15th week of feeding, mice were fasted with drinking freely during the period. For glucose tolerance tests, 12-hour-fasted mice were injected with d-glucose (2 g/kg body weight) intraperitoneally. For insulin tolerance tests, 6-hour-fasted mice were injected intraperitoneally with insulin (0.6 units/kg body weight). Blood samples were collected through the tail vein before insulin injection and at 15, 30, 60, and 120 min post-injection. The blood glucose concentration of mice was measured with blood glucose test strips.

### Serum Parameter Analysis

After 16 weeks of feeding, blood samples were collected from eyeballs removed from mice. After standing at 4°C for 6–8 h, the samples were centrifuged at 3000 r/min for 15 min, and then the serum was carefully moved to a clean EP tube. Triacylglycerol (TG), total cholesterol (TC), low-density lipoprotein cholesterol (LDL-C), high-density lipoprotein cholesterol (HDL-C), superoxide dismutase (SOD) and Malondialdehyde (MDA) was measured by biochemical assay kits, and the specific steps were carried out according to the manufacturer’s instructions. Furthermore, the levels of free fatty acids (FFA) in serum and bone homogenate, tumor necrosis factor-α (TNF-α) and interleukin-6 (IL-6) in serum were measured by ELISA kits.

### Micro-CT Analysis

A micro-CT scanner was utilized with the following parameters: 60 kV, 133 μA, 500 ms of single exposure time, 9 μm of scanning resolution, 0.5 degrees of scanning angle interval. Three-dimensional reconstruction images of trabecular bone were obtained by Hiscan Reconstruct software V1.0, and Trabecular bone volume/tissue volume (BV/TV), trabecular thickness (Tb. Th), Trabecular separation (Tb. Sp), Trabecular number (Tb. N) and other indicators were analyzed by Hiscan Analyzer software V1.0.

### Histomorphological Analysis

The femurs of mice fixed in paraformaldehyde were removed and placed in EDTA for decalcification at room temperature. The decalcified bone tissues were embedded in paraffin, sectioned and stained with hematoxylin and eosin (Wuhan Seville Biotech Technology Co., Ltd.). The closed slices were observed under an optical microscope and images were collected. About 10 fields were randomly selected, and the number of osteoclasts and fat cells were counted in each field.

### Western Blot

The bone tissue protein was extracted with Minute™ Bone tissue total protein extraction kit (Invent Biotechnologies, Inc., Eden Prairie, United States), according to the manufacturer’s instructions. The protein concentrations were determined by ultra-micro spectrophotometer (Implen, Munich, Germany) and were performed using Western blot analysis. After the protein samples were separated by electrophoresis in 10% SDS-PAGE, they were transferred to polyvinylidene fluoride (PVDF) membrane, blocked in skimmed milk powder at room temperature for 1 h, and incubated with AMPK, *p*-AMPK, ACC, *p*-ACC, CPT1, Nrf2, HO-1, GCLM, NQO1 and *β*-actin-specific primary antibodies overnight at 4°C, and the membrane was washed 3 times with 1×TBS-T for 5 min each time, then incubated with corresponding secondary antibodies at room temperature for 70 min. The protein intensity was detected by ECL developer and chemiluminescence imager, and the protein band density was quantified by Tanon Gis software.

### RNA Extraction and Quantitative Reverse Transcription-Polymerase Chain Reaction

Total RNA was extracted with a total RNA extraction kit. The cDNA was synthesized by PrimeScript RT Master Mix, an RNA reverse transcription kit. qRT-PCR was performed using SYBR Green I Master Mix according to the manufacturer’s instructions. After PCR was performed for 40 cycles, the expression of target gene mRNA relative to *β*-actin was calculated using the 2-ΔΔCt method. The primer sequences used in this study are shown in Supplementary Table S1.

### Statistical Analysis

The experimental results were expressed as the mean ± SD of at least three independent experiments. Statistical analyses were performed with SPSS 18.0 (Chicago, IL, United States). Data were tested for normality of distribution and homogeneity of variance before analyzing. Data that met normality and homoscedasticity were evaluated with one-way ANOVA. Otherwise, data were analyzed with the Kruskal-Wallis H test in the non-parametric tests. *p* < 0.05 was considered statistically significant.

## Results

### FA had No Significant Effect on the Body Weight of Mice Fed With HFD, but Affected Their Body Fat Ratio

**FIGURE 1:**
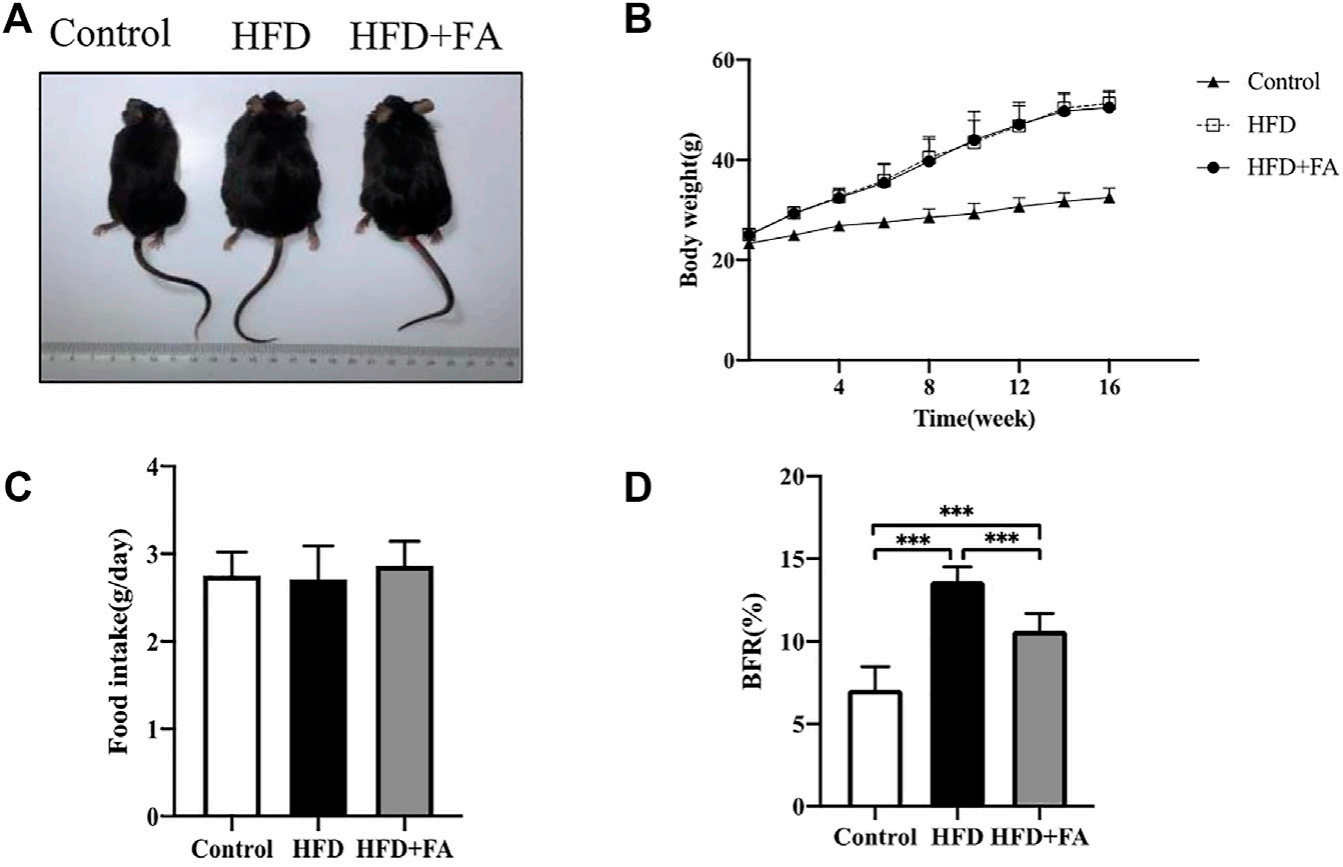
The effect of FA on the body weight and body fat of HFD fed mice. **(A)** Changes in body shape of mice in each group. **(B)** Changes in body weight of mice in each group. **(C)** Daily average food intake of mice in each group. **(D)** Body fat ratio of mice in each group. BFR(%) = adipose weight/body weight × 100%. The groin adipose, epididymal adipose, perirenal adipose and brown adipose were used in mice. All the data were expressed as mean ± standard deviation (*n* = 10). *p < 0.05; * *p < 0.01; * * *p < 0.001.

At the beginning of the experiment, the body weights of each group of mice were at the same level. During the experiment, the mice were weighed once a week. After 16 weeks of feeding, the weights of mice in the HFD group increased more than 20% compared with that in the control group. The results demonstrated that HFD induced obesity in mice, while FA intervention did not affect the weight gain of mice (Figures 1A,B), but could reduce the body fat ratio of mice fed with HFD (Figure 1C). Moreover, the food intake of mice was not affected by FA during the experiment (Figure 1D). It can be seen that FA intervention cannot reduce the body weight of high-fa-fed mice, but reduce their body fat ratio.

### FA Improves Bone Mineral Density and Bone Micro-architecture of Mice in a High-Fat Environment

**FIGURE 2:**
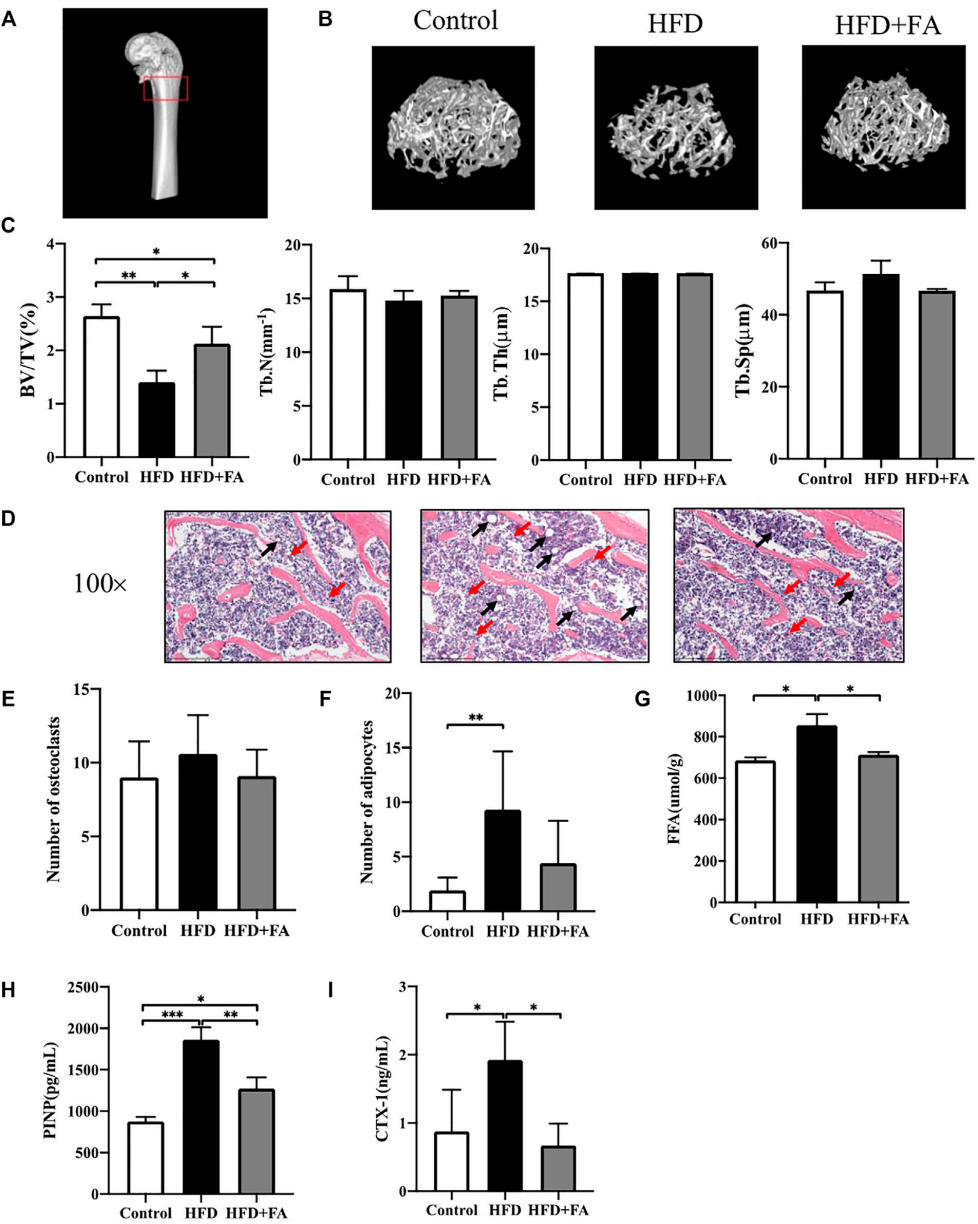
FA improves bone loss and destruction induced by high-fat diet in mice. **(A)** Schematic diagram of micro-CT analysis area. The area in the red box is the micro-CT analysis area. **(B)** Three-dimensional reconstruction images of the distal femur trabecular bone in each group. **(C)** Micro-CT bone parameters: bone volume/total volume (BV/TV), trabecular bone number (Tb.N), trabecular bone thickness (Tb.Th), trabecular bone spacing (Tb.Sp). **(D)** Representative H&E staining (100×) images of mouse distal femur sections. **(E,F)** Quantitative statistics of the number of osteoclasts and adipocytes in H and E staining images. Red arrows point to osteoclasts and black arrows point to adipocytes. **(G)** FFA level in bone homogenate. **(H,I)** PINP and CTX-1 levels in serum. All the data were expressed as mean ± standard deviation (*n* = 3). *p < 0.05; * *p < 0.01; * * *p < 0.001.

We next studied the effect of FA on the bone micro-architecture of mice. The 3D reconstruction images of the mouse distal femoral metaphysis clearly showed that the number of bone trabeculae in the HFD group was significantly reduced, and the bone micro-architecture was destroyed. However, FA intervention improved the quality and micro-architecture of the mouse distal femur trabeculae (Figure 2B). In addition, micro-CT analysis of distal femur showed that, in the HFD group, BV/TV and Tb. N were reduced, and Tb. Sp were significantly increased. These changes were attenuated after FA intervention. The effect of FA intervention on Tb. Th was not obvious (Figure 2C). Consistent with the results of micro-CT analysis, H and E staining of distal femoral sections confirmed that the number and thickness of femoral trabeculae of mice in the HFD group were reduced compared with those in the control group, and FA intervention attenuated these changes (Figure 2D). In addition, the number of osteoclasts and adipocytes in HE staining images was quantitatively analyzed, and the results showed that the number of osteoclasts and adipocytes in the HFD + FA group was reduced compared with that in the HFD group (Figures 2E,F). And FFA level in the bone homogenate of mice in the HFD group also decreased significantly after FA intervention (Figure 2G). We collected serum from the mice after 16 weeks of feeding and measured by ELISA the bone turnover markers crosslinked CTX-1 and PINP. We found that CTX-1 and PINP was significantly lower in the HFD + FA group compared to the untreated HFD group (Figures 2H,I). In summary, HFD can contribute to bone loss and destruction in mice, while FA intervention can improve bone mineral density and bone micro-architecture of mice in a high-fat environment.

### FA Attenuates Glucose and Lipid Metabolism Disorders Induced by HFD in Mice

**FIGURE 3:**
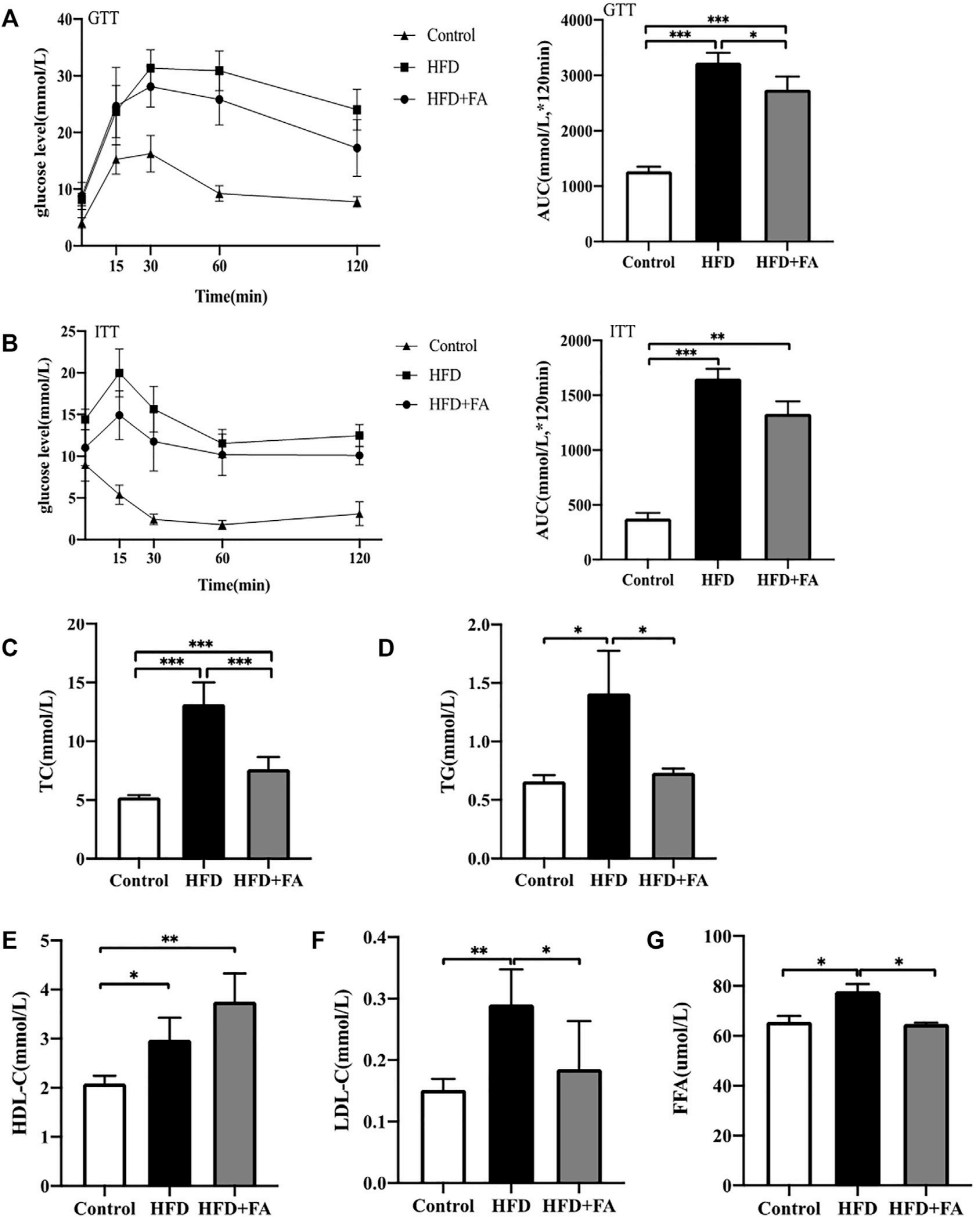
FA can improve lipid metabolism disorders induced by high-fat diet in mice. **(A)** Glucose tolerance test (GTT) curve and area under the curve (AUC). **(B)** Insulin tolerance test (ITT) curve and area under the curve. **(C–F)** TC, TG, HDL-C, LDL-C levels in serum. **(G)** Free fatty acid (FFA) level in bone homogenate. All the data were expressed as mean ± standard deviation (*n* = 3). *p < 0.05; * *p < 0.01; * * *p < 0.001.

In order to explore the effect of FA on glucose and lipid metabolism disorders induced by HFD, mice were intraperitoneally injected with glucose or insulin 1 week before sacrifice for glucose tolerance test or insulin tolerance test. We found that glucose tolerance of the HFD group was significantly lower than that of the control group, at the same time, the insulin sensitivity decreased, however glucose tolerance and insulin tolerance were improved after FA intervention (Figures 3A,B). Then we tested the effect of FA on the blood lipids of HFD-fed mice. The results showed that levels of TG, TC and LDL-C in serum of the HFD group were significantly higher than those in the control group, while levels of TG, TC and LDL-C in serum significantly decreased after FA intervention, and the level of HDL-C in serum increased (Figures 3C–F). In addition, we found that FA supplementation, to a certain extent, can reduce FFA levels in the serum of mice fed with HFD (Figure 3G). The above results indicate that FA intervention can attenuate glucose and lipid metabolism disorder induced by HFD in mice.

### Effect of FA on the Expression of Lipid Metabolism-Related Proteins in a High-Fat Environment

**FIGURE 4:**
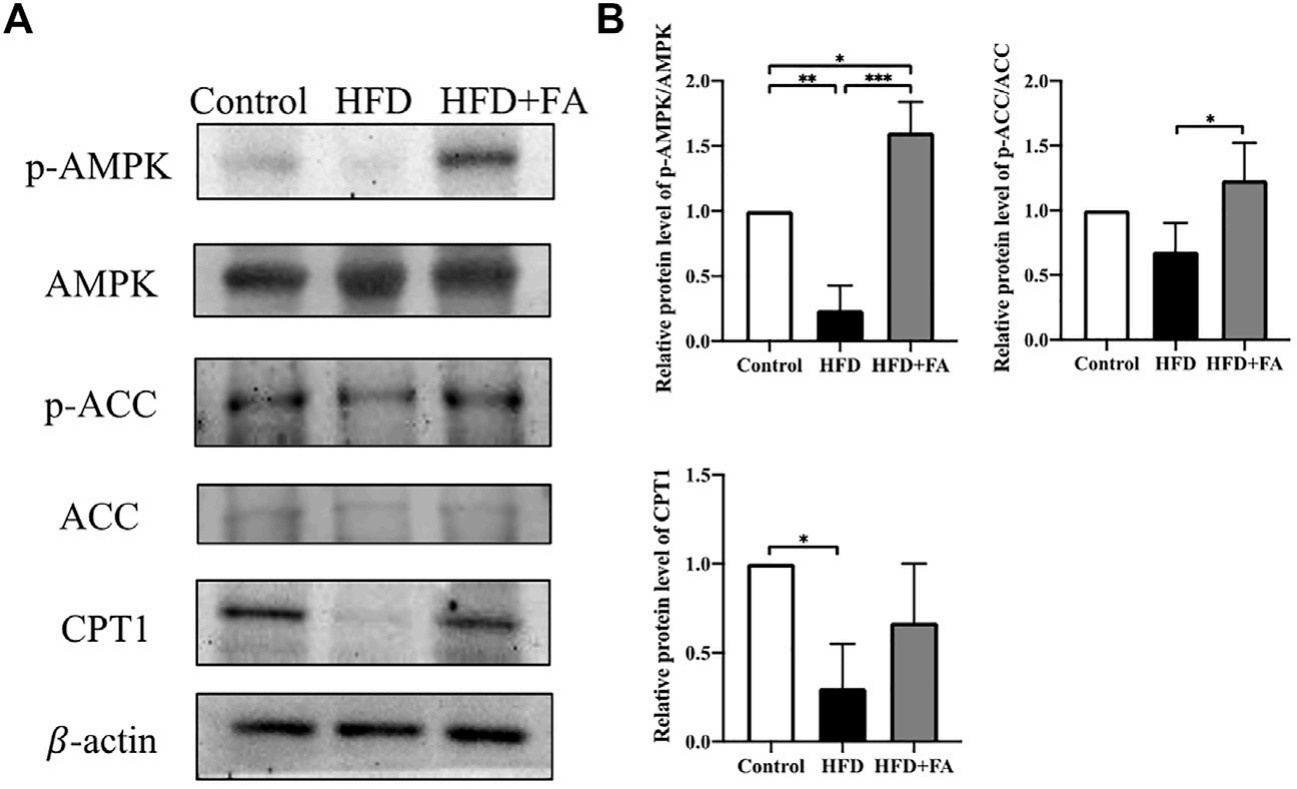
Effect of FA on the expression of lipid metabolism-related genes in a high-fat environment. **(A,B)** AMPK, *p*-AMPK, ACC, *p*-ACC, CPT1 protein levels were detected by Western blot. All the data were expressed as mean ± standard deviation (*n* = 3). *p < 0.05; * *p < 0.01; * * *p < 0.001.

In order to further explore the role of AMPK in FA affecting bone health in mice, we tested the protein levels of lipid metabolism-related genes in bone tissue of each group of mice. The results showed that compared with the Control group, the HFD significantly reduced the phosphorylation level of AMPK and its downstream protein ACC. Compared with the HFD group, FA treatment significantly increased the phosphorylation levels of AMPK and ACC, and promoted the expression of downstream CPT1(Figures 4A,B). These results indicate that FA may regulate the lipid metabolism of mice through the AMPK pathway, thereby protecting mice from bone loss.

### FA Improves Oxidative Stress and Chronic Inflammatory Reaction Induced by HFD

**FIGURE 5:**
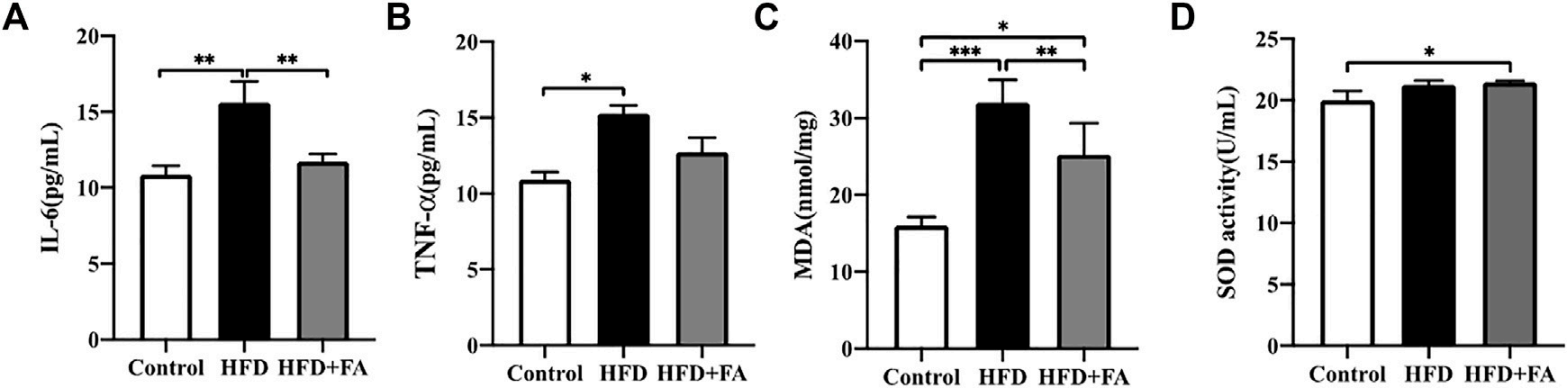
FA improves oxidative stress and chronic inflammatory reaction induced by high-fat diet. **(A,B)** Quantitative statistics of IL-6 and TNF-α levels in serum. **(C,D)** Quantitative statistics of MDA and SOD levels in serum. All the data were expressed as mean ± standard deviation (*n* = 3). *p < 0.05; * *p < 0.01; * * *p < 0.001.

TNF-α and IL-6 levels in the serum of mice were measured, and we observed the effect of FA intervention on chronic inflammation induced by HFD in mice. Compared with the control group, TNF-α and IL-6 levels in the serum of HFD group were significantly increased, while FA intervention can reduce TNF-α and IL-6 levels in the serum (Figures 5A,B). Then we studied the effect of FA on the oxidative damage induced by HFD in mice. We found that MDA level of the HFD group was significantly higher than that of control group, and reduced after FA intervention. But the effect of FA intervention on SOD level was not significant (Figures 5C,D). Therefore, we believe that FA intervention can improve oxidative stress and chronic inflammatory reaction levels of mice in a high-fat environment by reducing the levels of TNF-α and IL-6.

### FA Affects the Expression of Nrf2 and Phase II Enzymes

**FIGURE 6:**
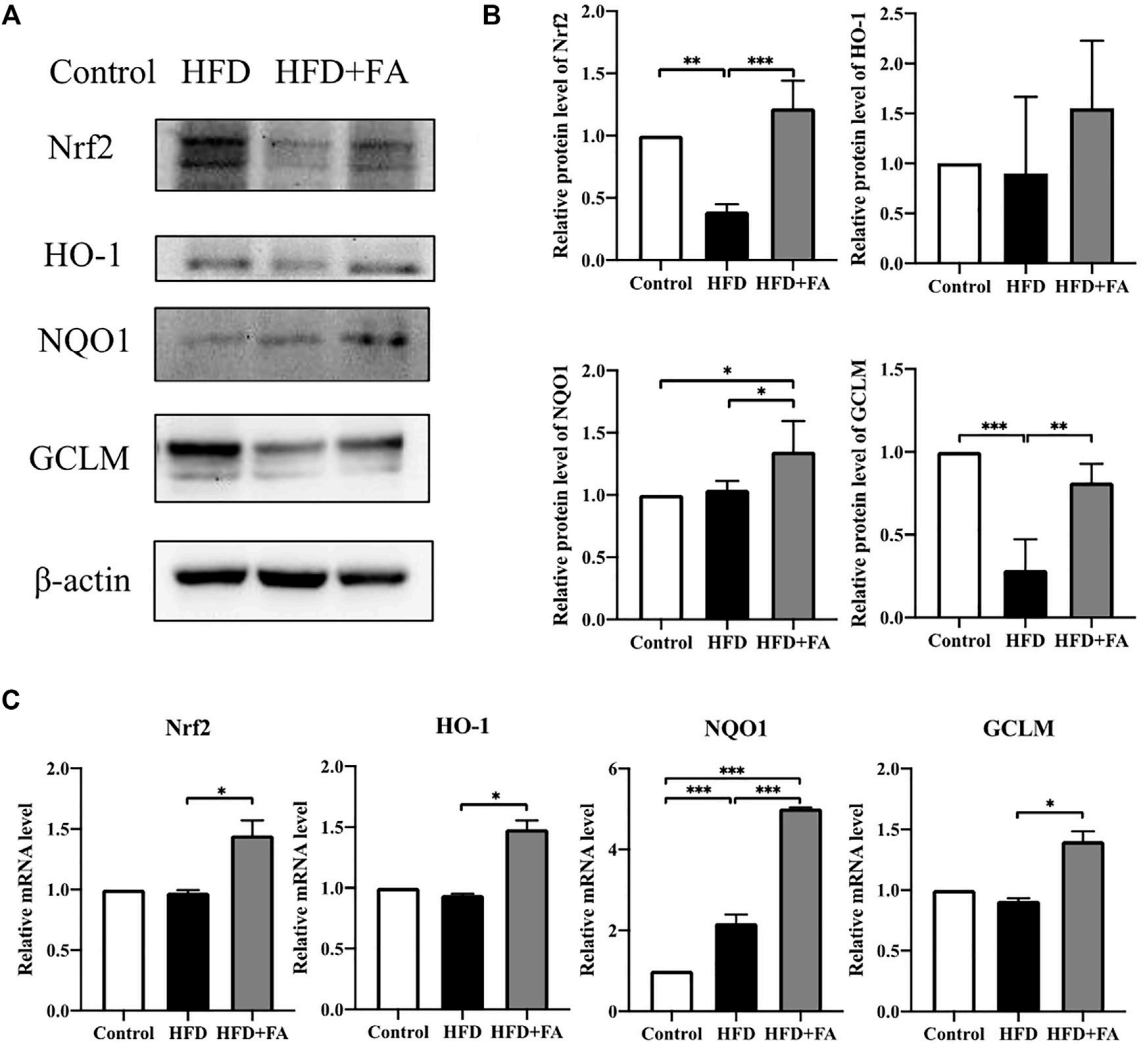
Effect of FA on the expression of Nrf2 and related enzymes in a high-fat environment. **(A,B)** Protein levels of Nrf2, HO-1, NQO1, and GCLM were detected by Western blot. **(C)** mRNA levels of Nrf2, HO-1, NQO1, and GCLM were detected by qRT-PCR. All the data were expressed as mean ± standard deviation (*n* = 3). *p < 0.05; * *p < 0.01; * * *p < 0.001.

In order to further explore the role of Nrf2 in FA affecting the bone health of mice, we tested the protein and mRNA expression levels of Nrf2 and phase II enzymes in the bone tissue of each group of mice. WB results showed that, compared with the HFD group, the expressions of Nrf2, HO-1, NQO1 and GCLM in the HFD + FA group were increased (Figures 6A,B), and qRT-PCR results were consistent with WB results (Figure 6C). These results suggest that the decreased expression of Nrf2 and phase II enzymes induced by HFD may be reversed by FA intervention.

## Discussion

**FIGURE 7:**
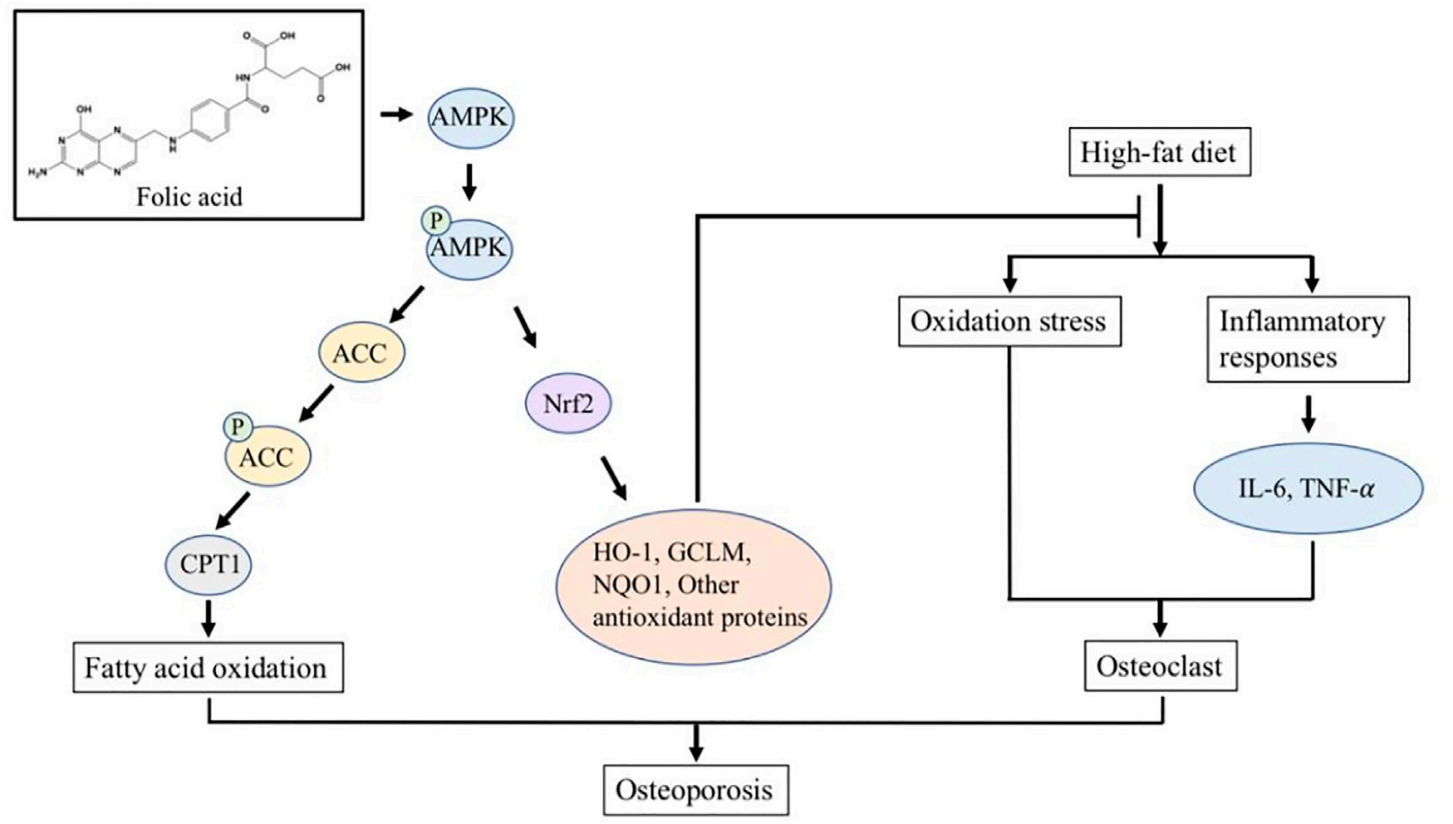
FA regulates lipid metabolism and oxidative stress in mice fed with high-fat diet by activating AMPK signaling pathway, thereby reducing osteoporosis induced by high-fat diet.

FA has been widely used in the treatment of megaloblastic anemia and the prevention of neural tube defects in neonates due to its extensive pharmacological activity and reliable safety. In this study, we constructed an HFD-induced osteoporosis model in C57BL/6 J mice to explore the effect of FA on HFD-induced osteoporosis and its molecular mechanism. Our results show that FA reduced the metabolic disorders induced by an HFD, including insulin resistance, hyperlipidemia and systemic inflammation, and reduced the level of oxidative stress. More importantly, we found that FA significantly improved bone mineral density and bone microstructure, and activated the AMPK signaling pathway, promoting the expression of lipid oxidation-related enzymes and antioxidant enzymes. In conclusion, we believe that FA can reduce osteoporosis induced by an HFD, which may be related to the regulation of lipid metabolism and oxidative stress levels by FA through the activation of the AMPK signaling pathway (Figure 7).

With the increasing availability of cooking oils and animal foods, it is predicted that 20% of adults worldwide will be obese by 2030 (Kelly et al., 2008). Several studies have shown that HFD is related to the development of obesity, and under the circumstances of obesity and osteoporosis are closely related to diseases (Shapses and Sukumar, 2012; Fabiani et al., 2019). Therefore, this study constructed an osteoporosis model by feeding mice with a high-fat diet. The model of osteoporosis induced by a high-fat diet refers to the influence of bone metabolism by ingesting a large amount of fat, thereby inducing the occurrence of osteoporosis. Obesity and osteoporosis have common characteristics, such as genetic predisposition, common progenitor cells. Both adipocytes and osteoblasts are derived from bone marrow mesenchymal stem cells. A long-term high-fat diet promotes adipogenesis and inhibits bone formation. In this study, the HFD significantly increased the weight of the mice, while FA did not affect weight gain (Figures 1A,B) or food intake (Figure 1C), but it could reduce body fat percentage (Figure 1D). It is suggested that the effect of FA on HFD-induced osteoporosis may not be through affecting the body weight but body fat percentage of high-fat-fed mice.

HFD has been reported to inhibit bone formation and enhance bone resorption (Tencerova et al., 2018; Roberts et al., 2021). Our study also proved that HFD could reduce bone mass and destroy bone microstructure in mice. The BV/TV of the HFD group was 46.7% lower than that of the Control group which was consistent with previous studies. The FA intervention can significantly improve the bone microstructure of mice on an HFD (Li et al., 2019; Lin et al., 2019). The specific manifestation is the increase in BV/TV, Tb. N and the connection density of trabecular bone after the intervention of FA, and the Tb. Th has no obvious change (Figures 2B,C), suggesting that the main effect of FA may be to increase the number of trabecular bone rather than thickening existing trabecular bone. Consistent with micro-CT analysis, the H&E staining of distal femur slices confirmed that the number and thickness of femur trabecular bone were reduced in the HFD compared with the control group, and FA attenuated these changes (Figure 2D). In addition, the number of osteoclasts was reduced and the number of adipocytes was increased in the HFD-fed mice after FA supplementation (Figures 2E,F). Meanwhile, FFA levels in bone homogenates were significantly reduced after FA supplementation (Figure 2G). Studies have shown that CTX-1 and PINP play an essential role in the pathogenesis of osteoporosis (Szulc, 2018). CTX-1 is an important osteoclast regulator, and the increase of CTX-1 can be used as a marker of the active function of osteoclast, which plays a vital role in osteoclast generation and osteoclast-mediated local bone loss (Eastell and Szulc, 2017). CTX-1 was significantly decreased after folic acid supplementation, but the effect on the number of osteoclasts was not obvious, suggesting that the effect of folic acid supplementation on osteoclasts may be more reflected in effect on their activity rather than the number. Type I collagen is the most abundant type of collagen in the human body and the only type of collagen in mineralized bone. The metabolites of its synthesis and decomposition can indirectly reflect the status of bone turnover. The content of PINP in serum reflects the ability of osteoblasts to synthesize collagen (Krege et al., 2014). The clinical study of Trovas GP (Trovas et al., 2002) showed that the bone resorption indexes NTX and CTX-1 decreased more significantly than the bone formation indexes BALP and PINP after the use of calcitonin, which can significantly reduce the bone turnover rate. Our results also show that FA reduced the serum CTX-1 and PINP levels of mice on an HFD (Figures 2H,I), and CTX-1 decreased more significantly than PINP, and the possible causes were analyzed: Generally, osteoporosis is caused by the increase of osteoclasts than osteoblasts, which breaks the balance between bone resorption and bone formation during normal bone reconstruction, resulting in osteoporosis. The bone formation index showed hyperfunction along with the increase of bone resorption index, which led to the increase of compensatory bone formation, which also led to the increase of bone formation index. However, the inhibition of this mechanism after short-term use of folic acid led to the decrease of bone metabolism index. It is possible that with the prolongation of folic acid supplementation, it will promote the proliferation of osteoblasts and increase bone formation indexes. Therefore, the results of this study cannot be considered contradictory at present.

Based on previous studies, we speculate that FA may affect the occurrence of osteoporosis by regulating lipid metabolism. Studies have shown that an HFD can cause glucose and lipid metabolism disorders in mice (Chen et al., 2019). The results of GTT and ITT show that the HFD group has impaired glucose regulation and reduced insulin hypoglycemic effect, indicating that HFD can induce glucose metabolism disorder and insulin resistance in mice. FA supplementation improved glucose regulation and insulin sensitivity in mice (Figures 3A,B). At the same time, FA can adjust the blood lipid level of HFD-fed mice (Figures 3C–F) and reduce the serum FFA content of mice (Figure 3G). Previous studies have shown that abnormal lipid metabolism and glucose metabolism are closely related to osteoporosis (Saoji et al., 2018; Veldhuis-Vlug and Rosen, 2018; Hu et al., 2019). Studies have found that long-term FA deficiency can lead to obesity, lipid metabolism disorders and glucose metabolism disorders, and appropriate FA supplementation can reduce the risk of dyslipidemia (Gargari et al., 2011; Dehkordi et al., 2016). Our results also show that FA intervention can reduce insulin resistance induced by an HFD and regulate lipid levels in mice, suggesting that FA has a positive effect on glycemic and lipid metabolism disorders. These beneficial effects are closely related to a reduction in lipid deposition in the body. We speculate that these changes may indicate that FA supplementation promotes lipid metabolism in the body, increasing additional lipid consumption.

Since FA can reduce lipid accumulation and improve bone microstructure in HFD-fed mice, we next try to explore the mechanism of FA. AMPK is a protein kinase that exists in the body, which can participate in the regulation of energy, glycolipid metabolism and mitochondrial homeostasis. AMPK is known as an energy regulator, and activation of AMPK is of great significance in treating obesity and obesity-related metabolic disorders (Carling, 2017; Garcia and Shaw, 2017). Many studies have shown that the activation of AMPK can control the overall lipid metabolism of cells by regulating ACC. ACC is a key enzyme that catalyzes lipid synthesis. Carnitine palmitoyl transferase 1 (CPT-1) is responsible for transporting fatty acids to mitochondria for oxidation, and ACC can convert acetyl-CoA to malonyl-CoA, an inhibitor of CPT-1. So the inactivation of ACC can increase fatty acid transport and subsequent oxidation, thereby reducing lipid synthesis (Vancura et al., 2018; Fadó et al., 2021). Our results show that the intervention of FA significantly increases the phosphorylation level of AMPK and ACC, and further up-regulates the expression of CPT1. This indicates that FA may activate AMPK, further promote the expression of CPT-1, and promote fatty acid oxidation to reduce lipid synthesis and lipid accumulation in the bone marrow cavity, thereby attenuating the osteoporosis induced by HFD.

Obesity is usually accompanied by chronic low-grade systemic inflammation, which leads to oxidative stress, and both inflammation and oxidative stress show harmful effects on the osteogenic differentiation of BMSCs (Napoli et al., 2017). The NF-κB signaling pathway mediated by TNF-α can regulate lipid metabolism, and IL-6 can promote systemic inflammation, leading to metabolic syndrome and abnormal lipid metabolism (Engin, 2017; Wang and He, 2018). In our study, FA significantly reduced the levels of TNF-α and IL-6 in the serum of mice, and reduced the inflammatory response (Figures 5A,B), suggesting that FA may regulate lipid metabolism by regulating TNF-α and IL-6. FA significantly reduced the expression of MDA. It increased the activity of the antioxidant enzyme SOD (Figures 5C,D), indicating that an HFD can induce lipid peroxidation in the body and reduce the activity of antioxidant enzymes in bone tissue, while FA supplementation improved the expression. Nrf2 is a transcription factor that protects cells and maintains bone metabolism from oxidative damage by regulating excessive antioxidant and cellular defense pathways (Dai et al., 2020). Under physiological conditions, Nrf2 exists in the cytoplasm and binds to kelch-like epichlorohydrin-related protein 1 (Keap1), and its activity is inhibited. When oxidative stress occurs in the body, phosphorylation of a series of protein kinases leads to the decoupling and translocation of Nrf2 and Keap1 into the nucleus, where Nrf2 binds with the antioxidant reaction element ARE to initiate the transcription and expression of AREs regulated antioxidant oxidase genes (Yamamoto et al., 2018). Several studies have shown that Nrf2 can be activated by AMPK (Lv et al., 2017; Duan et al., 2019). Nrf2 activation can activate downstream antioxidant enzymes, such as SOD, HO-1, NQO1, GCLM, etc., to play its antioxidant role and quickly and effectively remove excessive ROS produced in the body (Cuadrado et al., 2019). Our results showed that FA could up-regulate Nrf2 signaling pathway activity in the HFD-fed mice and promote the expression of downstream proteins HO-l, NQOl and GCLM, thereby activating antioxidant enzymes to mediate oxidative stress reduction (Figures 6A–C). These results suggest that FA may up-regulate the activity of antioxidant enzymes through the Nrf2 signaling pathway, protect cells from oxidative stress and inflammation-induced damage, and inhibit the activity of osteoclasts, thus contributing to the osteogenic differentiation of BMSCs, and thus reducing the high lipid-induced osteoporosis.

The present study has several limitations. First, the detailed mechanisms underlying the ability of FA to activate AMPK signalling have not been fully elucidated. Second, pharmacological parameters such as the optimal doses and treatment timing that are critical for clinical studies have not been well explored. Third, The potential mechanism of folic acid affecting skeletal health in mice was only preliminarily explored using animal models. Future work should target the molecular mechanisms underlying the protective effect of FA and explore its optimal administration in large animal models.

In summary, factors such as lipid metabolism disorders, inflammation, and oxidative stress damage together contribute to the formation and development of osteoporosis. This study shows that FA may have the effects of regulating metabolism, anti-inflammatory, and antioxidant. Therefore, FA may regulate lipid metabolism and oxidative stress in the HFD-fed mice by activating the AMPK signaling pathway, reducing osteoporosis induced by an HFD. Based on the positive effects of FA on the bone health of mice in this study, FA may provide a new idea for nutritional intervention strategies for osteoporosis caused by an HFD.

## Importance of the Study

Osteoporosis has become a worldwide epidemic. Among people over the age of 50, the incidence is one-third for women and one-fifth for men. It is estimated that the number of patients with osteoporosis worldwide will exceed 1 billion in 2020. Studies have found that obesity caused by a HFD will expand adipose tissue and cause chronic low-grade systemic inflammation. Ectopic adipose cells accumulate in the bone marrow cavity, which may impair bone regeneration and lead to osteoporosis. We proposed that FA may have a preventive effect on HFD-induced osteoporosis. Our research shows that FA can attenuated bone loss and improved bone microarchitecture in the HFD-fed mice. After the intervention of FA, the body fat rate of obese mice was reduced, and related metabolic disorders such as insulin resistance, hyperlipidemia, and systemic inflammation were alleviated. Thus, FA may provide a new idea for nutritional intervention strategies for osteoporosis caused by a HFD.

## Data Availability

The original contributions presented in the study are included in the article/Supplementary Material, further inquiries can be directed to the corresponding author.
