# Supplementary material for: Folic Acid Attenuates High-Fat Diet-Induced Osteoporosis Through the AMPK Signaling Pathway
Source: Front Cell Dev Biol. 2022 Jan 3;9:791880. doi: 10.3389/fcell.2021.791880 (PMC8762056; doi:10.3389/fcell.2021.791880)
Supplement: Supplementary file 1 [file Table1.pdf]

Supplementary Table 1. Primer sequences

Gene Name	Primer Sequence (5′ to 3′)	
	Forward	Reverse
Nrf2	CTGGCTGATACTACCGCTGTT	TGGAGAGGATGCTGCTGAAAG
HO-1	GCTGTGAACTCTGTCCAATGTG	TGTGTCAGGTATCTCCCTCCAT
GCLM	AAGAAGGCGGCTTGATGCTT	TCCAACTGAGCAGCAACACA
NQO1	GCCAATCAGCGTTCGGTATTAC	ACCTCCCATCCTCTCTTCTTCA
β-actin	GATCAAGATCATTGCTCCTCCTG	AGGGTGTAAAACGCAGCTCA
